# Antidiabetic and anti-obesity properties of a polyphenol-rich flower extract from *Tagetes erecta* L. and its effects on *Caenorhabditis elegans* fat storages

**DOI:** 10.1007/s13105-023-00953-5

**Published:** 2023-03-24

**Authors:** Sonia Núñez, Cristina Moliner, Marta Sofía Valero, Ahmed M. Mustafa, Filippo Maggi, Carlota Gómez-Rincón, Víctor López

**Affiliations:** 1 Department of Pharmacy, Faculty of Health Sciences, Universidad San Jorge, Villanueva de Gállego, Zaragoza, Spain; 2 Department of Pharmacology, Physiology and Legal and Forensic Medicine, Universidad de Zaragoza, Zaragoza, Spain; 3 Instituto Agroalimentario de Aragón, IA2, Universidad de Zaragoza-CITA, Zaragoza, Spain; 4 Chemistry Interdisciplinary Project (ChIP), School of Pharmacy, University of Camerino, Camerino, Italy; 5 Department of Pharmacognosy, Faculty of Pharmacy, Zagazig University, Zagazig, 44519 Egypt

**Keywords:** Edible flowers, *Caenorhabditis elegans*, Polyphenols, Advanced glycation End products, Lipase, Glucosidase, Fat accumulation

## Abstract

Diabetes mellitus (DM) is a metabolic disease characterized by a high blood sugar level that can cause severe complications to the organism or even death when not treated. However, certain dietary habits and foods may have beneficial effects on this condition. A polyphenolic-rich extract (containing hyperoside, isoquercitrin, quercetin, ellagic acid, and vanillic acid) of *Tageres erecta* L. (*T. erecta*) was obtained from yellow and orange flowers using an ethanolic Soxhlet extraction. These extracts were screened for antidiabetic and anti-obesity properties using in vitro and in vivo procedures. The capacity to inhibit the enzymes lipase and α-glucosidase, as well as the inhibition of advance glycation end-products (AGEs) was tested in vitro. *Caenorhabditis elegans (C. elegans)* was used as an obesity in vivo model to assess extracts effects on fat accumulation using the wild-type strain N2 and a mutant with no N3 fatty acid desaturase activity BX24. Extracts from both cultivars (yellow and orange) *T. erecta* presented in vitro inhibitory activity against the enzymes lipase and α-glucosidase, showing lower IC_50_ values than acarbose (control). They also showed important activity in preventing AGEs formation. The polyphenol-rich matrices reduced the fat content of obese worms in the wild-type strain (N2) down to levels of untreated *C. elegans*, with no significant differences found between negative control (100% reduction) and both tested samples (*p* < 0.05). Meanwhile, the fat reduction was considerably lower in the BX24 mutants (fat-1(wa-9)), suggesting that N3 fatty acid desaturase activity could be partially involved in the *T. erecta* flower effect. Our findings suggested that polyphenols from *T. erecta* can be considered candidate bioactive compounds in the prevention and improvement of metabolic chronic diseases such as obesity and diabetes.

## Introduction

Obesity and overweight are defined by abnormal or excessive fat accumulation that can impair health. Reports from World Health Organization (WHO) show that these conditions cause more deaths worldwide than underweight [76] and are linked to several complications and disorders such as diabetes, cardiovascular diseases, or cancer [3, 70].

Many obese suffer from metabolic complications such as type 2 diabetes (T2D). Diabetes mellitus (DM) is a metabolic disease characterized by high blood sugar level that can cause severe complications to the organism or even death when not treated. About 422 million people worldwide suffer from this condition, mainly T2D, and 1.5 million deaths are directly attributed to this disease each year [52]. Both the number of cases and the prevalence of diabetes have been steadily increasing over the past few decades [53]. Uncontrolled diabetes can cause metabolic imbalance leading to acute complications that may require immediate medical attention. Hyperglycemia (elevated levels of glucose in blood) sets the stage for protein glycation [25, 67] and the production of reactive oxygen species (ROS) [73], which in turn may lead to chronic conditions requiring constant monitoring and treatment.

Glucosidase enzymes catalyze the hydrolysis of starch to simple sugars. In humans, these enzymes aid the digestion of dietary carbohydrates and starches to produce glucose for intestinal absorption, which in turn, leads to an increase in blood glucose levels. Inhibiting the function of these enzymes in patients with T2D may reduce hyperglycemia. Lipase inhibition is one of the most widely studied mechanisms for the determination of the potential efficacy of natural products as anti-obesity agents. Orlistat is the registered drug for the treatment of obesity and its mechanism of action works through an irreversible inhibition of pancreatic and gastric lipase [28, 57]. However, there are important side effects derived from the use of orlistat mainly in the gastrointestinal system [22]. Advanced end glycation products (AGEs) are a group of compounds created by the nonenzymatic glycation of proteins, lipids, or nucleic acids endogenous or exogenously. Although they are known for being created during hyperglycemia periods, they can also be formed during high-temperature cooking and food processing [65]. Dietary AGEs are partially absorbed and represent an important source of these molecules in the organism. AGEs contribute to the pathogenesis of age-related diseases, such as diabetes or cardiovascular diseases [12], accumulating in certain damaged organs such as the kidneys, the retina, and blood vessels [25], damaging them. In T2D patients, the endothelial dysfunction leads to other cardiovascular risk factors [67]. From a pharmacological point of view, it has been shown that oral drugs such as metformin or pioglitazone reduce AGEs formation; aminoguanidine (AMG) was also one of the first AGEs formation inhibitors studied [9] and has been effective in animals but its studies in humans were discontinued due to side effects and low efficacy [69]. A new term has been made up to describe the joint occurrence of T2D and obesity: “diabesity” [29], acknowledging that an excess of body fat is the major cause of T2D. The dietary intervention has been recognized to play a significant role in the prevention and management of T2D since moderate weight loss has been shown to improve blood pressure, glycemic values, insulin resistance, and dyslipidemia [16]. In recent years, preventive medicine has been consolidated as one of the most important health strategies, and several authors have expressed that the ingestion of natural products with high polyphenol content can play a beneficial role in the prevention and improvement of lifestyle-related diseases such as obesity and diabetes [18, 45, 46]. Our diet comprises a wide range of plant foods (vegetables, fruits, legumes, nuts, herbal teas) being edible flowers a culinary ingredient that is gaining attention currently. *Tageres erecta* L. (*T. erecta*) is an edible flower native to Mexico widely used in gastronomy but also as a medicinal and ornamental species. It is also cultivated to extract lutein, which has many applications as a food additive or nutritional supplements [6]. The previous research done over this edible flower has mainly focused on antiaging and antioxidant properties [10, 26, 47]; however, antidiabetic or anti-obesity properties have been less explored [68]. The in vivo studies previously performed with this edible flower are found in the fields of antiparasitic [11, 54], antioxidant [4, 47], and anti-inflammatory activity [44]. However, no in vivo research has previously been done for anti-obesity or antidiabetic properties with whole flower extracts. In the field of obesity, one of the easiest models to work with is *Caenorhabditis elegans* (*C. elegans*) [60, 81]. *C. elegans* is a model organism widely used for the evaluation of functional foods, nutraceuticals, and bioactive compounds because approximately 60–80% of the human genes have their homologue in the *C. elegans* genome; therefore, physiological processes and important metabolic pathways are maintained [81]. The fatty acids in *C. elegans* are stored mainly in lipid droplets [61]. Its transparency allows us to observe and quantify the lipid disposition by measuring the intensity accumulated by a wide range of dyes [19, 33]. Previous research has shown that phenolic compounds such as ellagic and vanillic acid, some of the main polyphenols found in *T. erecta*, can reduce the lipid content of *C. elegans* [2].

Given the rich polyphenolic content characterized in *T. erecta*, the purpose of the current study was to assess the antidiabetic and anti-obesity activity of *T. erecta* using in vitro procedures and for the first time in an in vivo *C. elegans* model.

## Materials and methods

### Reagents

α-glucosidase from *Saccharomyces cerevisiae*, 4-nitrophenyl α-D-glucopyranoside (pNPG), lipase from porcine pancreas, 4-nitrophenyl butyrate (NPB), Nile Red, fructose, acarbose, and aminoguanidine bicarbonate (AMG) were purchased from Sigma-Aldrich (St. Louis, MO, USA). Na_2_HPO_4_, NaH_2_PO_4_, Tris–HCl, CaCl_2_, KH_2_PO_4_, and Na_2_N_3_ were purchased from Panreac Quimica (Castellar del Vallès, Barcelona, Spain). Bovine serum albumin (BSA) was purchased from Santa Cruz Biotechnology, Inc., and orlistat from Acofar (Terrassa, BCN, Spain). Ethanol, methanol, and formic acid were purchased from Carlo Erba Reagents (Val de Reuil Cedex, France).

### Samples and polyphenolic extraction

Edible flowers of two cultivars of *T. erecta* with yellow and orange petals were purchased from Innoflower SL. Whole fresh flowers were cut into small pieces and extracts were prepared with a soxhlet apparatus using ethanol at an extraction temperature between 80 and 85 °C for 4 h. The solvent was removed with a rotatory evaporator, and the resulting extracts were stored in the dark at − 20 °C.

### Analysis of polyphenols by HPLC–MS/MS

The quantification of 38 bioactive molecules (37 of them are phenolic compounds) was carried out using a modified version of a previously described method [48]. The extracts were re-dissolved at a concentration of 5 mg/ml with ethanol. The HPLC–MS/MS investigations were carried out with an Agilent 1290 Infinity series and a Triple Quadrupole 6420 (Agilent Technology, Santa Clara, CA, USA) and linked to an electrospray ionization (ESI) source that operated in negative and positive ionization modes. Using Optimizer Software, the MS/MS parameters of each standard were optimized using flow injection analysis (FIA). The bioactive compounds were separated in gradient elution mode on a Phenomenex Synergi Polar–RP C18 column (250 mm × 4.6 mm, 4 µm) using a mixture of water and methanol as solvents A and B, respectively, both with 0.1%, formic acid. For column protection, a Polar RP security guard cartridge preceded the column (4 mm × 3 mm ID). The mobile phase composition was made up of the following components: 0–1 min, isocratic condition, 20% B; 1–25 min, 20–85% B; 25–26 min, isocratic condition, 85% B; 26–32 min, 85–20% B. A 0.2-μm polyamide filter was used to filter all solutions and solvents. The injection volume was 2 μL and the flow rate was kept at 0.8 mL/min. The temperature of the column was set to 30 °C, and the drying gas temperature in the ionization source was set to 350 °C. The flow rate of the gas was set to 12 L/min, the capillary voltage was 4000 V, and the nebulizer pressure was 55 psi. The peak areas were integrated for quantitation after detection in the dynamic-multiple reaction monitoring (dynamic-MRM) mode. Each analyte’s most abundant product ion was employed for quantification, while the other ions were used for qualitative analysis. The results were expressed as mg/kg of extract.

### In vitro bioactivity assays

#### Inhibition of α-glucosidase

The capacity of polyphenolic extracts from *T. erecta* to inhibit α-glucosidase was measured in a 96-well microplate reader at 405 nm [30]. Each well contained a 50 µL sample and 100 µL enzyme (1 U/mL) solved in buffer (12.5 mM Na_2_HPO_4_, 3.3 mM NaH_2_PO_4_; pH = 6.9). After 10 min of incubation at room temperature, 50 µL pNPG (3 mM) was added and incubated at 37 °C for 15 min (absorbance readings took place every 5 min since the addition of substrate). Control wells contained 50 µL of solvent.

The inhibition was calculated using the following formula (Eq. 1):

1 $$Inhibition\;\left(\%\right)=\left[\frac{\left(Abs\;control-Abs\;sample\right)}{Abs\;control}\right]x100$$

#### Inhibition of pancreatic lipase

Lipase inhibition was quantified in 96 well plates. Forty microliters of extract solution (serial dilutions) was mixed with 40 µL of the enzyme (2.5 mg/mL in 0.1 M TRIS base buffer with 5 mM CaCl_2_, pH = 7.0) previously centrifugated at 2000 g for 7 min, and 20 µL of substrate solution (10 mM of p-NPB). After incubation for 15 min at 37 °C, absorbance was read at 405 nm. Orlistat was used as a positive control. The percentage of lipase inhibition was calculated using Eq. 1.

#### Advanced glycation end products formation inhibition

Inhibition of AGEs formation by the polyphenolic-rich extracts was measured by fluorescence in 96 black well-plates [64, 65]. A total of 50 μL of BSA solution (10 mg/mL), 80 μL of 0.1 M phosphate buffer (containing sodium azide 3 mM and pH = 7.4), 50 μL of fructose solution (0.5 M), and 20 μL of sample extracts (serial dilutions) were mixed. After incubating for 24 h at 37 °C, plates were analyzed at an excitation wavelength of 355 nm and emission wavelength of 460 nm. Aminoguanidine (AMG) was used as a positive control. The inhibition of AGEs formation was calculated using Eq. 1.

### In vivo* assays using C. elegans*

#### *C. elegans* strains and maintenance conditions

*C. elegans* strain N2, Bristol (wild-type), and strain BX24 (*fat-1(wa-9))* were provided by the Caenorhabditis Genetics Center (CGC, University of Minnesota, Minneapolis, MN, USA). Nematodes were grown and maintained on nematode growth medium (NGM) at 20 °C using *Escherichia coli* OP50 and *Escherichia coli* OP50-GFP bacteria as food sources also obtained from the CGC. For all experiments, synchronized worms N2 and BX24 were obtained by an alkali-bleaching method [39].

#### Chemotaxis assay

To evaluate whether the *T. erecta* extracts were attractant or repulsive substances, a chemotaxis assay was performed using a previously described method [42]. A 55 mm petri dish is divided into four quadrants, two “T” for the test substance and two “C” for the control. L4 stage N2 worms were washed three times in M9 to eliminate bacteria, and approximately 50–100 worms were placed in the center of the plate right before starting the assay. After 60 min, worms in each quadrant were recorded. This assay was performed three times in triplicates.

The chemotaxis index, which would be comprised between − 1.0 (repulsing) and + 1.0 (attracting), was calculated using the following formula (Eq. 2):

2 $$Chemotaxis\;Index\;\left(CI\right)=\left[\frac{\left(Worms\;in\;Test\;quadrants-Worms\;in\;Control\;quadrants\right)}{Total\;of\;worms}\right]$$

#### Evaluation of T. erecta flower extracts on *C. elegans* fat storages and lipid droplets analysis

An obese *C. elegans* model was created after exposing the worms to an excess of 5% glucose in the nematode growth medium (NGM) and was performed in two *C. elegans* strains: wild-type N2 and mutant BX24 (*fat-1(wa-9)*).

Different condition agar plates were prepared by adding the testing substances directly to the NGM: 5% glucose as a positive control (Gluc), 5% glucose and 6 µg/mL orlistat as negative control (orlistat), yellow *Tagetes* extract (YT), orange *Tagetes* extract (OT), and plates with only NGM as a non-obese control (NGM).

Every condition studied was exposed to glucose excess except for control, which represents the normal nematode growth and development. Both extracts were tested at three concentrations 500, 250, and 125 µg/mL. As a negative control substance, orlistat was used at a concentration of 6 µg/mL [43], the fat-reduction obtained by this drug compared to the obese worm (positive control) will be considered the maximum effect (100% reduction).

The effects of the extracts on *C. elegans* fat storages were studied by Nile Red staining and fluorimetry, and the obtained value of fluorescence intensity per area is the relative value that allows us to express the fat content of *C. elegans.*

Synchronized L1 *C. elegans* (at least 300 individuals per condition) were grown for 48 h at 20 °C until they reach the L4 stage under different dietary conditions: plates with NGM as a control diet or NGM supplemented with 5% glucose and different doses of the studied extracts (500, 250, and 125 µg/mL).

The nematode fat content was measured by Nile Red staining image quantification, and the straining process was followed as previously described [19]. Nile Red dyeing allows to observe the lipid accumulation in intracellular droplets of L4 stage worms thanks to the *C. elegans* transparency. These dyed lipidic globules emit fluorescence when exposed to ultraviolet light (Nikon Intensilight C-HGFI). A total of 30–40 worms per condition were captured with a Nikon camera attached to an inverted microscope Nikon Eclipse TS100 after exposure to UV lightning using a GFP filter that captures at 395 nm excitation and 508 nm emission wavelength. All worms were photographed at 100 × magnification and 20 s of exposure time. Images were analyzed using the image processing program ImageJ to obtain the relative fluorescence per area value of each worm. Additionally, using the same images and processing program, the lipid droplet numbers and sizes of each condition were studied.

#### *E. coli* ingestion quantification assay

The effect of the extracts over the feeding rate was studied through quantification of fluorescent bacteria detectable in the nematode after treatment exposure using the following described method. Same conditions as the previous assay were adopted except for the bacteria feeding the *C. elegans* and a new treatment condition containing only the polyphenolic extracts. A strain of OP50 that contains a GFP plasmid (pFPV25.1) was used, this stain is fluorescent when exposed to UV light at wavelength 480 nm excitation and 571 nm emission. Synchronized L1 *C. elegans* (at least 300 individuals per condition) were grown for 48 h at 20 °C until they reach the L4 stage under the conditions mentioned in the previous assay. NGM supplemented with the extracts at a concentration 500 µg/mL was included as a new condition for this assay. Fluorescence was quantified using a spectrophotometer and 96 black-well plates. After worms reach the L4 state, they were washed with PBS to remove remnants of *E. coli* and at least 40 individuals per condition were introduced on each well in triplicate, using PBS as blank. Values of fluorescence showed the amount of bacteria OP50-GFP ingested after exposure to the different conditions.

### Pharyngeal pumping assay

Synchronized L1 *C. elegans* were grown for 48 h at 20 °C until they reach the L4 stage under the conditions mentioned in the previous assays, using only the higher dose of extracts (500 µg/mL). Videos of 30 s were recorded for each individual, and the pharyngeal pumping was measured by counting the rhythmic contraction of the pharynx during that time-lapse. Each worm was counted three times and data averaged. This assay was repeated independently three times.

### Statistical analysis

GraphPad Prism 6.0 (GraphPad Software, San Diego, CA, USA) was used for statistical analysis. All experiments were performed in triplicates on different days, and their results were plotted as mean ± standard error means (SEM).

Non-linear regression fit with one phase decay was performed to fit the curves, and IC_50_ was calculated with a 95% confidence interval; Student-test was used to detect differences between both samples. To measure the fluorescence intensity per area of the *C. elegans* after Nile Red straining, ImageJ 1.53c was used, and to analyze the data analysis of variance (ANOVA), Tukey’s multiple comparisons was performed.

## Results

### HPLC–MS/MS analysis of polyphenols

The polyphenolic content of the ethanolic extracts was obtained using a HPLC–MS/MSS analysis and is shown in Table 1. Extraction yields for the two plants were 3.17% (mass of extract/ mass of fresh flowers) for orange flowers and 3.45% for the yellow ones. A total of 11 phenolic acids and 16 flavonoids were identified and quantified. The total phenolic content was higher in the yellow extract being 10,511.78 mg/kg of dry extract opposed to the 8101.54 mg/kg of dry extract of the orange flower. Regarding the phenolic acids, ellagic acid was the most abundant in both cultivars followed by vanillic acid. The most abundant flavonoid compounds found were hyperoside, isoquercitrin, and quercetin, following the previous bibliography [10, 49].

**Table 1:** Content (mg·kg^−1^ of dry extract) of bioactive compounds in yellow and orange *T. erecta* extracts analyzed by HPLC–MS/MS

No	Compound	Yellow *T. erecta*	Orange *T. erecta*
Phenolic acids			
1	Gallic acid	240.13	316.71
2	Neochlorogenic acid	64.50	38.65
3	Chlorogenic acid	279.71	262.72
4	p-hydroxybenzoic acid	47.46	51.55
5	Caffeic acid	334.08	150.42
6	Vanillic acid	1069.06	791.11
7	Syringic acid	59.72	70.26
8	p-coumaric acid	105.00	123.65
9	Ferulic acid	232.18	96.76
10	3,5-dicaffeoylquinic acid	5.84	7.51
11	Ellagic acid	6295.36	5230.49
Flavonoids			
(A) Anthocyanins			
12	Delphinidin 3,5 diglucoside	243.98	136.12
13	Petunidin-3-glucoside	0.24	0.36
(B) Flavonols			
14	Rutin	2.31	1.56
15	Isoquercitrin	311.04	152.90
16	Quercitrin	17.27	7.37
17	Kaempferol-3-glucoside	31.14	17.69
18	Quercetin	459.86	191.49
19	Isorhamnetin	72.21	57.46
20	Hyperoside	491.72	293.63
21	Kaempferol	25.04	17.36
(C) Flavan-3-ols			
22	Catechin	0.18	1.42
23	Procyanidin B2	44.12	27.47
24	Procyanidin A2	22.11	22.11
(D) Dihydrochalcones			
25	Phloridzin	10.95	8.81
26	Phloretin	0.19	0.05
(E) Flavanones			
27	Hesperidin	46.41	25.92
Total phenolic content		**10,511.78**	**8101.54**

### In vitro bioactivity on α-glucosidase, pancreatic lipase, and AGEs formation

**Fig. 1:**
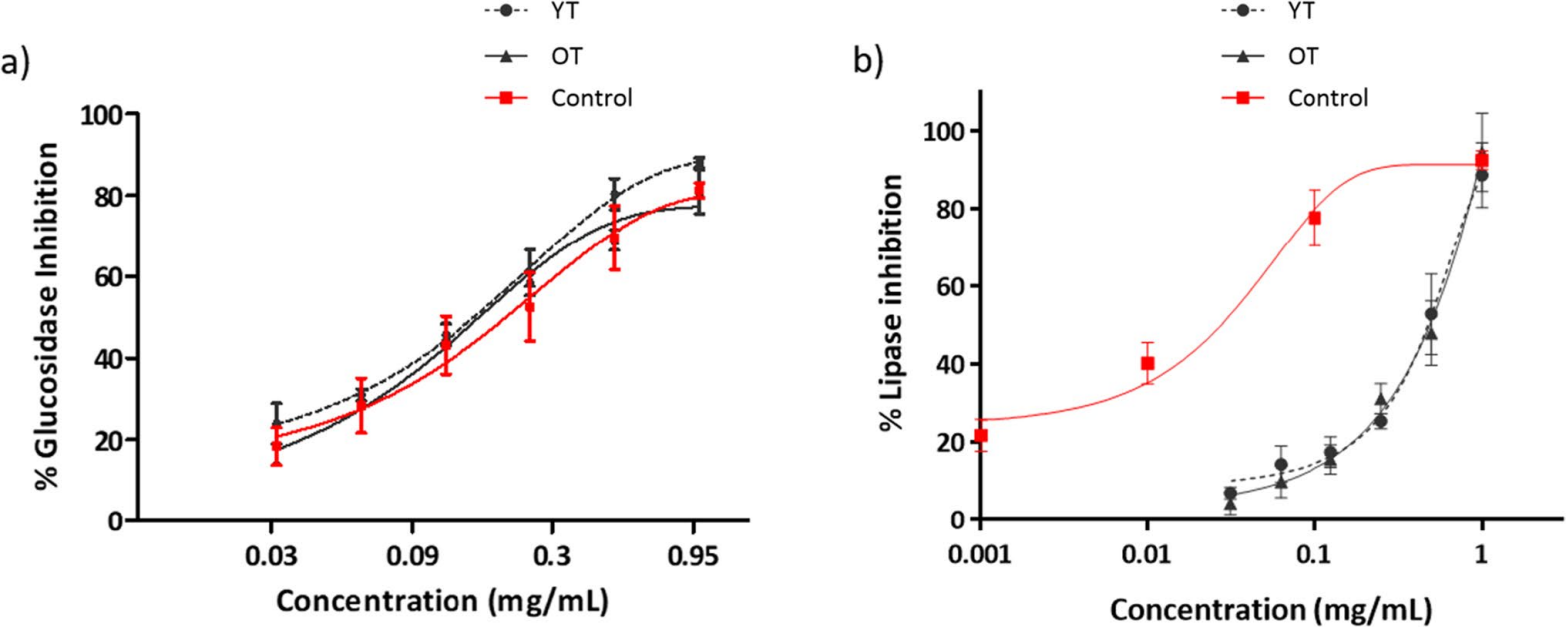
Inhibition of α-glucosidase (**a**) and pancreatic lipase (**b**) by yellow and orange extracts of *T. erecta*. Acarbose (**a**) and orlistat (**b**) were used as a positive control. Results show mean ± SEM (*n* = 3). The studied range of doses was 31.25–1000 µg/mL in both assays. No significant differences were found between the two flower extracts using Student *t*-test statistical analyses in both the assays. YT, yellow *Tagetes*; OT, orange *Tagetes*

*T. erecta* flower extracts had the ability to inhibit in a dose-dependent manner the enzymes α-glucosidase and pancreatic lipase (Fig. 1). With respect to inhibition activity against α-glucosidase enzyme, both *Tagetes* polyphenol-rich extracts showed a higher inhibition than the control acarbose (Fig. 1a). The yellow extract was the most active with IC_50_ of 201.83 ± 38.89 µg/mL; meanwhile, the orange extract had IC_50_ of 275.86 ± 11.89 µg/mL. In the lipase enzyme inhibition assay, both extracts obtained similar results, without significant differences between the two different cultivars. The orange extract showed a lower IC_50_ value than the yellow extract, 473.75 ± 59.96 µg/mL vs. 479.46 ± 59.05 µg/mL (Fig. 1b).

**Fig. 2:**
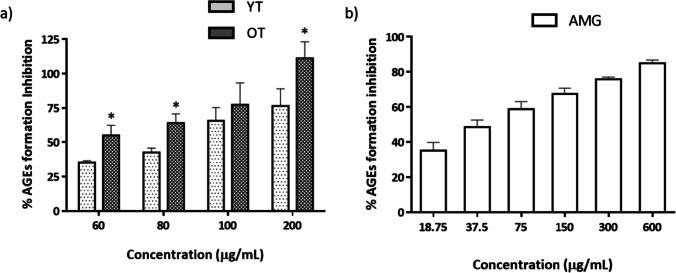
Inhibition of advanced glycation end-products (AGEs) by **a** yellow and orange *T. erecta* extracts and **b** AMG positive control. Data are presented as mean ± SEM (*n* = 3). YT, yellow *Tagetes*; OT, orange *Tagetes.* *p < 0.05 vs YT

As shown in Fig. 2, both samples had an important activity against the AGEs formation. Orange *T. erecta* extract showed lower IC_50_ (47.19 ± 17.71 µg/mL) in the inhibition of protein glycation with respect to yellow extract (70.61 ± 6.53 µg/mL) and control, AMG (77.82 ± 6.86 µg/mL). These results showed significant differences between the two extracts (Table 2).

**Table 2:** Calculated IC_50_ (µg/ml) ± standard error for each matrix in the inhibition of the in vitro assays. No significant differences were found between the two flower extracts using Student *t*-test statistical analyses except for AGEs inhibition

Extract	α-glucosidase inhibition	Lipase inhibition	AGEs formation inhibition
Yellow cultivar	201.83 ± 38.89	479.46 ± 59.05	77.82 ± 6.86
Orange cultivar	275.86 ± 11.89	473.75 ± 59.96	47.19 ± 17.71**
Control	297.21^a^ ± 15.81	27.68^b^ ± 13.31	70.61^c^ ± 6.53

(**p < 0.01). ^a^Acarbose, ^b^orlistat, ^c^AMG

### In vivo assays on* C. elegans*

#### Lipid droplets quantification in N2 wild-type and BX24 mutant strains

**Fig. 3:**
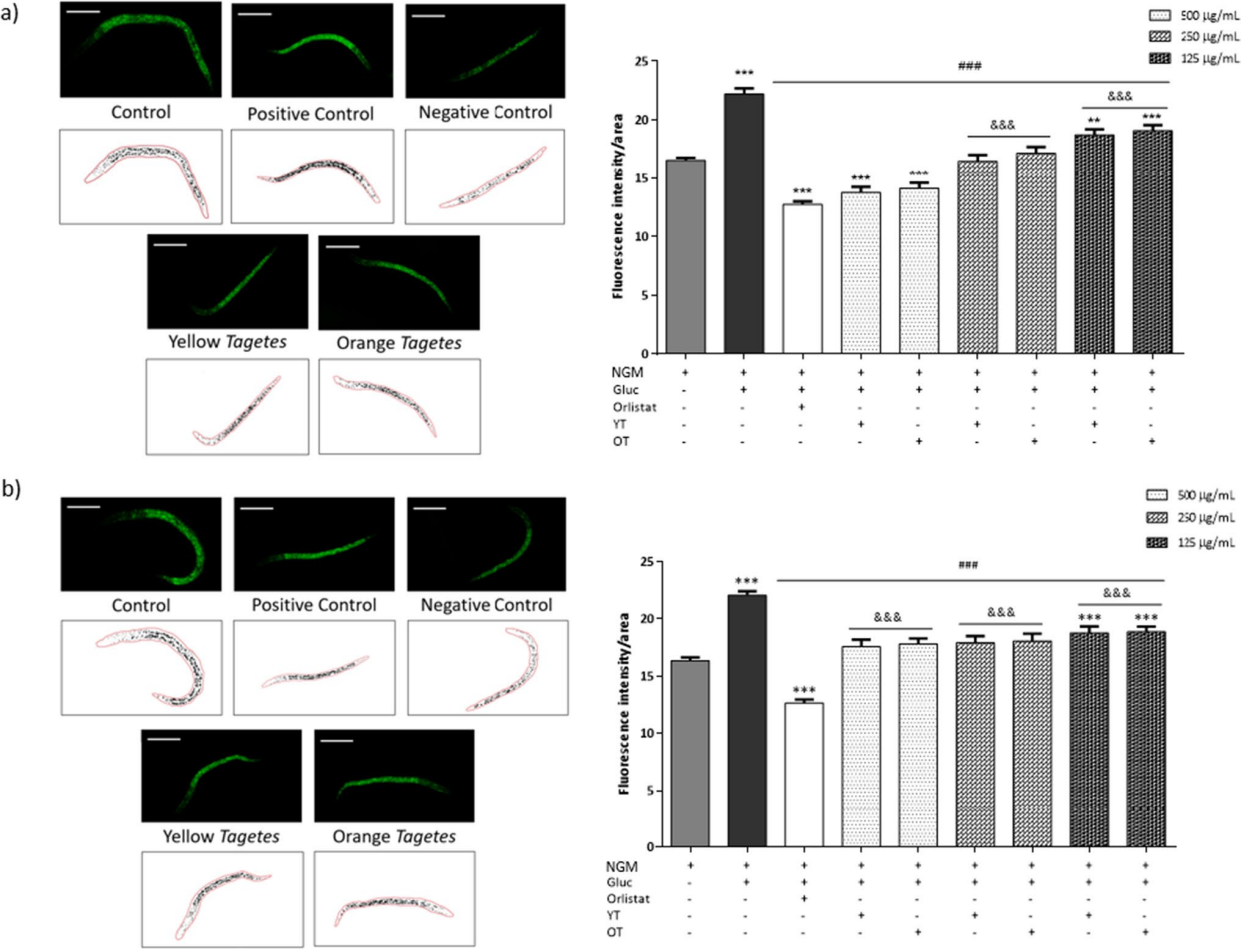
Fluorescence images, lipid droplets profile, and total lipid droplet quantification of *C. elegans*, **a** N2 strain and **b** BX24 mutant strain. Fluorescence images of *C. elegans* were taken after exposing the worms to the different conditions and after filtering with Nile Red and exposure to ultraviolet light; below, lipid droplets are highlighted. Only worms treated with extract (500 µg/mL) images are shown. Scale bar = 120 µm. Histogram showing the relative values of lipids in *C. elegans* wild type (N2) and mutant strain (BX24) obese model after being exposed to different concentrations of *T. erecta* extracts. Results are represented as mean ± SEM. (*n* = 60–90 worms). Three independent biological replicates were performed. NGM, control; Gluc, glucose 5% (positive control); Orlistat, glucose 5% + orlistat 6 µg/mL (negative control); YT, yellow *Tagetes*; OT, orange *Tagetes*. **p < 0.01, ***p < 0.001 vs NMG; ^###^*p* < 0.001 vs Gluc; ^&&&^*p* < 0.001 vs Gluc + orlistat

The extracts showed a dose-dependent reduction on the wild type *C. elegans* fat storages. As Fig. 3a shows, in N2 worms treated with glucose only (obese worms), there was a significant increase in lipid content compared to control worms (NMG), treated with culture medium only. Orlistat, an anti-obesity reference drug, significantly reduced lipid droplets in the obese worms even below the lipid content of control worms. In the same way, the highest concentration of *T. erecta* extracts tested (500 µg/mL) also reverted the increase in fat content in the obese worm, without significant differences between the drug and the extract. Considering the effect of orlistat as a 100% reduction in lipid droplet formation, 500 µg/mL of the extracts produced a reduction in fat content similar to orlistat (89.44% and 85.68% reduction for yellow and orange *T. erecta* respectively). At the concentration of 250 µg/mL, *T. erecta* produced a significant decrease in fat content in the obese worm similar to that of the control worms (*p* > 0.05). The reduction in fat content was 60.47% and 57.54% for yellow and orange respectively. Finally, the lowest concentration of extract, 125 µg/mL, showed the least reduction in fat deposits compared to obese worms, with a fat reduction of 36.45% and 34.13% for yellow and orange *T. erecta*, respectively. No significant differences were found at the same concentrations between the two different cultivars (*p* > 0.05).

**Fig. 4:**
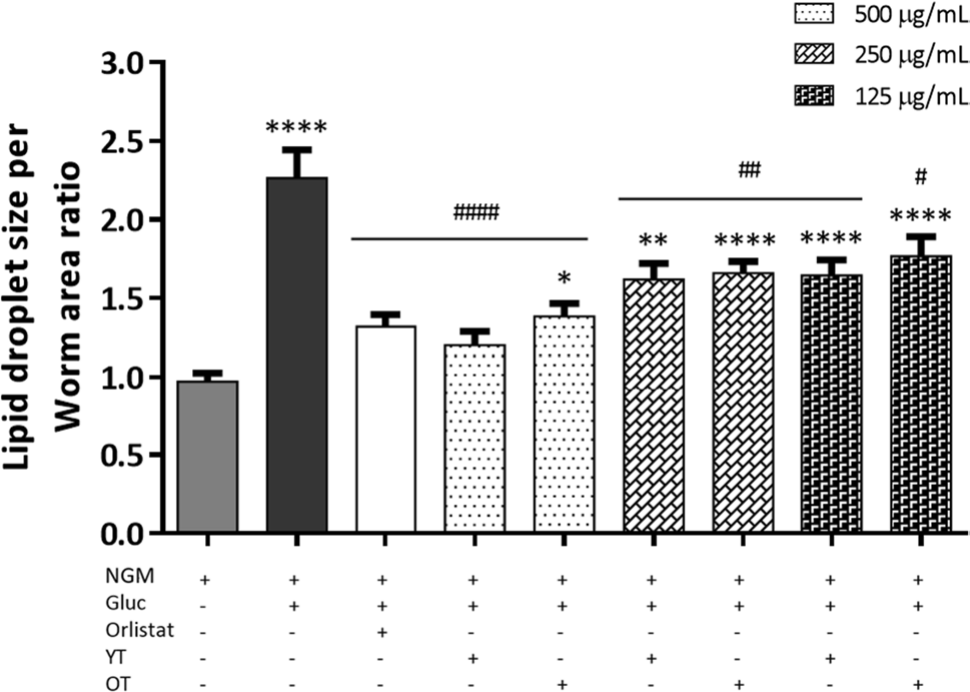
Lipid droplet average size per worm area ratio of *C. elegans* N2 strain. Results are represented as mean ± SEM. (*n* = 50–60 worms). NGM, control; Gluc, glucose 5% (positive control); Orlistat: glucose 5% + orlistat 6 µg/mL (negative control); YT, yellow *Tagetes*; OT, orange *Tagetes*. *p < 0.05, **p < 0.005, ****p < 0.0001 vs NMG; ^#^*p* < 0.05, ^###^*p* < 0.005, ^####^*p* < 0.0001 vs Gluc

To further assess the reduction in lipid content, lipid droplet size from every condition was studied in the N2 wild-type strain [37]. The lipid droplet profile can be seen in Fig. 3 below the corresponding fluorescence images. Exposure to high concentrations of glucose as mentioned before produces a series of damages to *C. elegans*, one of the most noticeable is the reduction of worm size. To relativize the obtained data, lipid droplet size was expressed as a ratio between lipid size per worm area (Fig. 4), since the exposure to glucose significantly reduces the worms’ areas. Regarding that ratio, untreated NGM worms showed the lowest value of 0.97 ± 0.05, followed by orlistat and the higher dose of both *Tagetes* extracts being 1.32 ± 0.08 orlistat, 1.20 ± 0.09 yellow *Tagetes*, and 1.38 ± 0.08 orange *Tagetes*. At the concentration of 250 µg/mL, the ratio values obtained were 1.62 ± 0.10 for yellow and 1.66 ± 0.07 for orange; meanwhile, the lowest concentration 125 µg/mL obtained values of 1.64 ± 0.10 and 1.77 ± 0.12 for yellow and orange *Tagetes* respectively. The highest ratio was obtained by the obese condition, excess of glucose, with a value of 2.26 ± 0.18. No differences were found between orlistat and every dose of flower extract tested (*p* > 0.05).

The BX24 mutant strain has no N3 fatty acid desaturase activity. In this mutant, the fat levels obtained by control worms, obese worms, and worms treated with glucose and orlistat showed similar results to those obtained by worms of the wild-type strain N2. However, there were differences in lipid content compared to N2 worms when treated with the extracts of *T. erecta*. All three doses of extracts showed a similar reduction in fat content, slightly significant, between 47 and 32%, compared to BX24 obese worms (Fig. 3b). However, in this case, no doses of extract produced the orlistat-like effect.

#### *E. coli* ingestion quantification

**Fig. 5:**
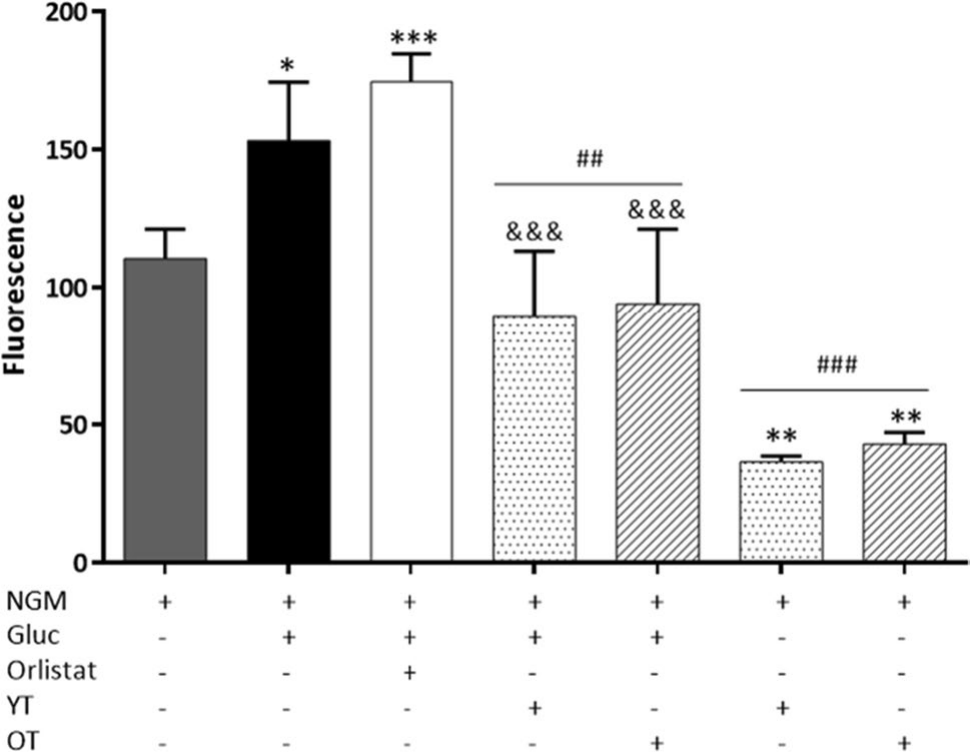
Fluorescence of N2 *C. elegans* after exposure to different conditions and being fed with *E. coli* OP50-GFP for 48 h. Results are represented as Mean ± SEM (*n* = 30–40 worms). *p < 0.05, **p < 0.01, ***p < 0.001 vs NGM; ^##^*p* < 0.01, ^###^*p* < 0.001 vs Gluc; and ^&&&^*p* < 0.001 vs Gluc + orlistat. Values with different superscripts indicate significant difference at *p* < 0.05. NGM, control; Gluc, glucose 5% (positive control); Orlistat, glucose 5% + orlistat 6 µg/mL (negative control); YT, yellow *Tagetes*; OT, orange *Tagetes*

The fluorescence emitted by the *E. coli* ingested by the worms after being exposed to the different conditions can be observed in Fig. 5. Excess glucose significantly increased *E. coli* intake in obese worms compared to control worms (NMG). This increase was significantly reversed to control worm ingestion levels by *T. erecta* extracts at the highest dose (500 µg/mL).

The ingestion reduction was observed in control worms (non-obese) when treated with both *T. erecta* extracts only, with a decrease in *E. coli* intake of 67.02% and 61.14% for the yellow and orange cultivars, respectively, compared to control ingestion (NGM). No significant differences were found between the two cultivars. Finally, orlistat treatment did not reduce the *E. coli* intake.

#### Pharyngeal pumping assay

**Fig. 6:**
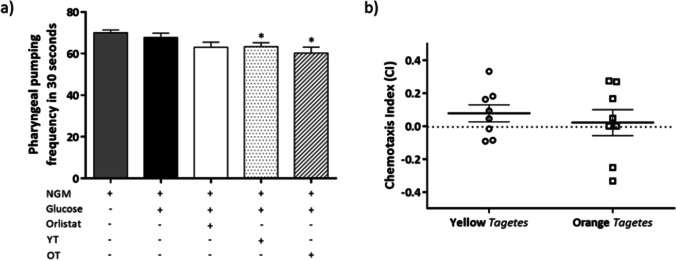
**a** Pharyngeal pumping of N2 *C. elgans* after exposure to different conditions for 48 h. Results are represented as mean ± SEM. *p < 0.05 vs NGM. NGM, control; Gluc, glucose 5% (positive control); Orlistat, glucose 5% + orlistat 6 µg/mL (negative control); YT, yellow *Tagetes*; OT, orange *Tagetes*. **b** Chemotaxis index (CI) of N2 *C. elegans* exposed to *T. erecta* extracts. Results are represented as mean ± SEM. No significant differences were found between the two extracts

The pharyngeal contraction of the worm’s pharynx exposed to the different conditions can be observed in Fig. 6a. The nematodes exposed to *T. erecta* extracts at the highest dose (500 µg/mL) significantly reduced the rate of pharynx pumping compared to the control untreated worms (NGM), being the reductions of 9.56% and 13.99% for the yellow and orange cultivars respectively. There were no differences found between the NGM control and the excess glucose or the orlistat treatment (*p* > 0.05).

#### Chemotaxis assay

The CI obtained by the flower extracts can be observed in Fig. 6b, being values of 0.07796 ± 0.05 for yellow *Tagetes* and 0.02187 ± 0.08 for orange *Tagetes.* No significant differences were found between the two extracts, and neither comparing them to the neutral value of CI zero.

## Discussion

As obesity and overweight rates are increasing worldwide, leading to severe health complications such as diabetes and cardiovascular diseases, preventing the progress of these conditions is becoming key to their management and control. Diet can play a major role in the development of cardiovascular diseases, and the ingestion of natural products with high polyphenol content can play a beneficial role in the improvement of lifestyle-related diseases such as obesity and diabetes [18, 45, 46]. *T. erecta*, an edible flower native to Mexico, has been used both in gastronomy and medicine. The edible flowers of *T. erecta* have shown in vitro antioxidant, anti-inflammatory, and anti-aging properties [4, 10, 26, 44, 47]. Here, it is shown for the first time the in vitro and in vivo, in a *C. elegans* model, antidiabetic and anti-obesity effect of *T. erecta* whole flower extracts.

The characterization of our extracts from *T. erecta* yellow and orange flowers showed a rich content in polyphenols being quercetin, hyperoside, isoquercitrin, ellagic acid, and vanillic acid the main compounds characterized (Table 1). Polyphenols are secondary metabolites found in plants with multiple properties and uses, and they represent the most abundant natural compounds with antidiabetic properties [18] within plants and nature.

The in vitro activities obtained by the extracts (Figs. 1 and 2) are in line with the previously reported activities of the main polyphenols found in *T. erecta* extracts. Thus, hyperoside also known as quercetin-3-O-galactoside has been reported in many in vitro bioactive properties such as α-amylase [36] and α-glucosidase inhibition [39, 41], antioxidant activity [39], and inhibition of AGEs formation [5]. Ellagic acid is also a natural antioxidant with anti-inflammatory and antidiabetic properties [17]. Isoquercitrin, quercetin, and vanillic acid have also been shown to be good α-glucosidase inhibitors [35, 78] and antioxidants [50, 71].

Quercetin extracted from *T. erecta* has also proved to have anti-lipolytic and antidiabetic activity [27, 74], but it is the first time that lipase inhibition activity has been reported from the whole flower extract. The IC_50_ values for α-glucosidase inhibition (Table 2) obtained with these extracts were lower than other *T. erecta* extracts previously studied [75] and showed no significant difference between the two cultivars.

The differences found in the AGEs formation inhibition between the yellow and orange extracts might be related to the pigment compounds that give color to the petals such as carotenes [62], since orange flowers have higher carotenoid content in their composition than yellow flower extracts based on previous studies [80]. Moreover, carotenoids have proven to own protective effects over cardiovascular diseases [21].

*C. elegans* exposure to high levels of glucose leads to ROS formation which is also related to AGEs formation [58], decreases the lifespan of the worm [13, 23, 32], and produces several damages such as apoptosis and mitochondrial dysfunction [1] as well as increase fat accumulation [81]. A previous study showed that *T. erecta* extracts have protective effects under lethal oxidative stress on *C. elegans* as well as increase its lifespan [47]. One of the main compounds found in the extracts, hypersoside, has shown both in vitro and in vivo (mice) anti-inflammatory activity against damages produced by high-glucose exposure [31] and has also been tested in vivo for hyperglycemic activity [72] and reduction of ROS levels [24], both related to T2D complications.

The tested extracts reduced the levels of fat in N2 wild-type strain to values of the control drug orlistat (concentration 500 µg/mL) and values of the non-treated control (concentration 250 µg/mL). The fat reduction is also displayed by the lipid droplet profile (Fig. 4). The lipid droplet size per worm’s area ratio showed that obese worms had the biggest size of droplets formed as lipids tend to fuse and aggregate, going in correlation with having the highest amount of fat. On the opposite side, orlistat-treated worms showed small droplets which had no statistical difference to the values obtained by the flower extracts at any concentration, value also correlating to the fat levels (Fig. 3a) that showed orlistat-treated worms. This activity of the extracts is so potent that the negative effects caused by a high glucose diet could potentially be reverted. The obese worms treated with orlistat increased the bacteria intake but showed the lowest fluorescence values after lipid staining with Nile Red. Previous works revealed that orlistat’s mechanism of action is based on the inhibition of pancreatic lipase [28, 57] and cholesterol esterase [66], meaning it does not affect the nematode’s feeding. Those statements are reinforced by the result obtained in the pharynx pumping assay, where orlistat did not change the contraction rate compared to neither control worms (NGM) nor obese worms (excess glucose). The polyphenolic extracts showed activity inhibiting the pancreatic lipase, but the IC_50_ values were high. However, as shown through the *E. coli* ingestion and pharynx pumping assays (Figs. 5 and 6a), the extracts reduced the amount of bacteria ingested in both obese and non-obese models and obtained values of fat storages equal to orlistat at the higher concentration, and equal to non-treated worms at medium concentration. There are reports of natural compounds like kahweol that have previously proved to reduce the food intake in *C. elegans* [20]; this diterpene was able to reduce fat accumulation by reducing the pharynx pumping rate of the worm and therefore reducing the amount of *E. coli* able to ingest. As mentioned before, the bacteria ingestion is also reduced after exposure to the extracts, affecting therefore the lipid accumulation.

Regarding the BX24 mutant worms, the fat content was reduced but with a lower effect than the N2 wild-type worm. All the concentrations tested obtained a reduction in the fat content without significant differences among them, and without a difference to concentration 125 µg/mL on N2 strain. Although there were no differences among the tested concentration effects, the higher concentration tested (500 µg/mL) of both extracts reduced fat content to levels of control *C. elegans*. This could mean that the extract mechanism of action over the *C. elegans* fat storages might be partially related to the gene mutated, the activity of N3 fatty acid desaturase, since there is a reduction, but it is limited. N3 fatty acid desaturase, also known as omega-3 desaturase, is one of the 7 desaturase enzymes that *C. elegans* own for the biosynthesis of long-chain polyunsaturated fatty acids [8]. Our results suggest that additional mechanisms could be involved in the observed effects.

There are many ways in which lipid storages in the *C. elegans* organism can be modified. There have been reports of polyphenols reducing fat content in *C. elegans* by reducing the food intake [20], increasing lipolysis [2], inhibiting lipogenesis [79], and through different pathways [7, 55, 59, 77]. Regarding the compounds found in our ethanolic extracts, quercetin has proved to protect *C. elegans* from glucotoxicity [14], vanillic and ellagic acid reduced fat content in the nematode [2], and the latter increases its lifespan [56].

One of the main pathways in which polyphenols exert their action to prolong the lifespan of *C. elegans* and protect them from oxidative stress [40, 51] is through the gene *daf-16* that mediates the insulin/insulin-like growth factor signaling pathway (IIS), pathway related to lipid metabolism and management [15] that can be targeted to reduce fat accumulation in *C. elegans* [34]. Serotonin is known to play a key role in the feeding of *C. elegans* [63], and the serotoninergic system can be also targeted to induce fat loss in the nematode [38]. Based on results obtained after the exposure of the polyphenol-rich *T. erecta* extracts to N2 wild-type and BX24 mutant *C. elegans*, we could speculate that the fat reduction these extracts exert in the worms might be mediated partially by the gene mutated in the transgenic nematode *fat-1(wa-9)* in addition to the reduction of food intake previously stated.

## Conclusions

This work has shown that the two flower extracts from *T. erecta* rich in polyphenols have anti-diabetic and anti-obesity properties by reducing lipid levels and bacteria intake in obese *C. elegans*. This effect could be due in part to the *(fat-1(wa-9))* gene, although more studies should be carried out to identify the action pathway of these extracts, as well as to identify the main bioactives responsible for these effects. Considering their antidiabetic and anti-obesity capacities, these flower cultivars could be considered candidate products for the prevention and improvement of chronic metabolic diseases such as obesity and diabetes.

## Abbreviations

*AGEs*: Advanced glycation end products
*AMG*: Aminoguanidine
*CI*: Chemotaxis index
*DM*: Diabetes mellitus
*NGM*: Nematode growth medium
*ROS*: Reactive oxygen species
*SEM*: Standard error mean
*T2D*: Type 2 diabetes
